# A Single-Chip High-Voltage Integrated Actuator for Biomedical Ultrasound Scanners †

**DOI:** 10.3390/s19235063

**Published:** 2019-11-20

**Authors:** Chin Hsia, Yi-Chi Hsiao, Yen-Chung Huang

**Affiliations:** 1 Department of Electrical Engineering, National Central University, Taoyuan 32001, Taiwan; 2 Industrial Technology Research Institute, Hsinchu 31040, Taiwan

**Keywords:** biomedical ultrasound, high-voltage pulse driver, ultrasonic transducer/actuator, slew-rate, low-power supply

## Abstract

This article presents a high-voltage (HV) pulse driver based on silicon-on-insulator (SOI) technology for biomedical ultrasound actuators and multi-channel portable imaging systems specifically. The pulse driver, which receives an external low-voltage drive signal and produces high-voltage pulses with a balanced rising and falling edge, is designed by synthesizing high-speed, capacitor-coupled level-shifters with a high-voltage H-bridge output stage. In addition, an on-chip floating power supply has also been developed to simplify powering the entire system and reduce static power consumption. The electrical and acoustic performance of the integrated eight-channel pulse driver has been verified by using medical-grade ultrasound probes to acquire the transmit/echo signals. The driver can produce pulse signals >100 Vpp with rise and fall times within 18.6 and 18.5 ns, respectively. The static power required to support the overall system is less than 3.6 mW, and the power consumption of the system during excitation is less than 50 mW per channel. The second harmonic distortion of the output pulse signal is as low as −40 dBc, indicating that the integrated multi-channel pulse driver can be used in advanced portable ultrasonic imaging systems.

## 1. Introduction

Biomedical ultrasound imaging, which is relatively fast, inexpensive, portable and radiation-free compared to computed tomography (CT), X-ray and magnetic resonance imaging (MRI), has become one of the most popular modalities for clinical examinations [1]. Currently, in addition to conventional B-mode and Doppler ultrasonic modality, tissue harmonic imaging is becoming an important medical evaluation tool for echographic medical systems because of its characteristics of easy integration into conventional ultrasound scanners and higher lateral resolution, lower side lobes, and reduced sensitivity to clutter and off-axis distortions [2,3,4]. The principle of tissue harmonic imaging is to transmit an ultrasonic wave at a certain fundamental frequency and receive an echo at the harmonics of the frequency (usually the second harmonic) that is generated by the nonlinear propagation of waves in the tissue [5]. The method needs to control the transmitter of the ultrasound scanner well, since the high-frequency harmonics generated in the ultrasonic transmitted pulses easily interfere with the harmonics generated in the tissue in the signal propagation process, degrading the image quality. In addition, the detected echo signal generated by the nonlinearity reaction of the propagating medium is easily affected by other harmonic leakages in the system, including the limited transducer bandwidth and nonlinearity of the receiver itself [2,3,4,5]. Since the linearity and bandwidth requirements of transmitters used in tissue harmonic imaging are different from those in traditional B-mode ultrasonic applications, a bipolar pulse sequence generated by a high-voltage (HV) bipolar pulse driver with low static content and less harmonic distortions is more suitable for driving ultrasound actuators and/or transducers in such applications than a unipolar pulse generator [6,7,8]. However, the problem of power consumption and various harmonic leakages still exists in real system applications and, therefore, more advanced techniques are required to improve overall performance of bipolar pulse sequences [9,10]. In order to improve the characteristics of transmitted pulse signals for tissue harmonic imaging, additional considerations are required for the ultrasound pulse driver design. For instance, an increase in output pulse frequency and slew-rate allows the pulse driver to actuate the medical transducer at a higher oscillation frequency, which can be used to increase the resolution of the ultrasound image. Moreover, bipolar pulses with the same on/off duty cycles and rising/falling edges have lower even harmonic contents that have to be within the power of, at least, 20 dB less than that of the fundamental signal from the acoustic measurement when using a hydrophone [11,12]. In order to provide sufficient signal-to-noise ratio for the harmonic imaging, harmonic signals generated by the drivers themselves should be as low as possible. Further, for increasingly demanding portable solutions, multi-channel pulse drivers require integrated circuits with minimum off-chip components to reduce overall sizes and the associated power dissipations [13,14,15,16,17,18]. Some previous works have used multiple voltage-level architectures to reduce harmonic distortion [19,20,21,22], and these techniques have typically required several voltage sources, adding design complexity. In [23], we demonstrated a single channel pulse driver integrated circuit (IC) for actuating the transducers in ultrasound imaging systems. However, due to the device breakdown voltage limit, the output voltage of the reported pulse driver in [23] was within 80 Vpp, resulting in insufficient excitation energy for driving the medical transducer. In addition, the architecture employed multiple off-chip voltage sources, which increased the overall size and power consumption of the ultrasound transmitter. Based on the requirements of the ultrasound driver for harmonic imaging system applications, an eight-channel ultrasound pulse driver was developed with a higher output voltage level using the complementary metal oxide semiconductor–silicon on insulator (CMOS–SOI) technology, and was presented in [24]. Due to the limited page space, only the basic measurement results of the circuit were reported in [24]. In this paper, we give an in-depth description of the CMOS–SOI based high–voltage ultrasound driver design that is specifically designed with an emphasis on linearity, operating voltage, bandwidth and overall power consumption. In particular, we will present the transmit signal characteristics, the low-power high-speed circuit design principles and the procedures of synthesis of the single-chip ultrasound transmitter system.

This paper is organized as follows. Section 2 reviews the characteristics of the pulse excitation signals required in a harmonic imaging system. Section 3 describes the detailed design and circuit-level implementation of a high-voltage ultrasound pulse driver. In Section 4, experimental results are given for driving medical-grade piezoelectric transducers (PZTs) with the designed driver. The conclusion is provided in Section 5. 

## 2. Excitation Signals for Harmonic Imaging System

**Figure 1:**
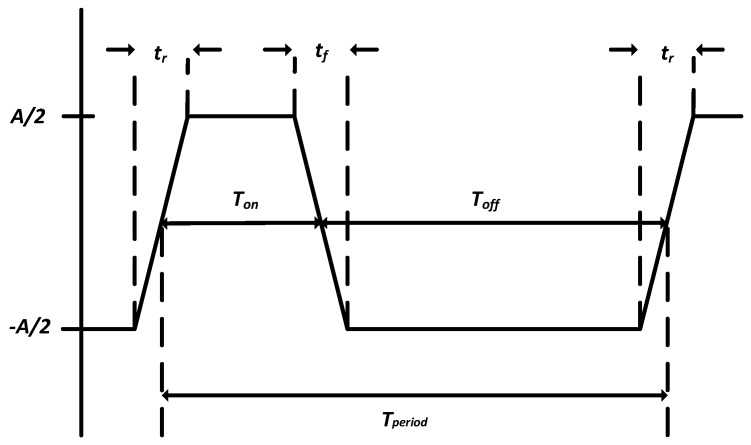
A typical trapezoidal waveform with a limited rise/fall time.

Since high-voltage excitation signals such as square waves or trapezoidal waveforms can efficiently drive ultrasonic transducers, harmonic components of such waveforms need to be considered in order to achieve low harmonic output for certain applications. Given the nature of these ideal waveforms, Fourier analysis makes it easier to display their spectrums and explore the possibility of generating such waveforms [25]. In this section, the basic spectrum of the high–voltage pulse waveform is analyzed specifically from the perspective of the harmonics of the pulse wave as a design standard for the low second harmonic pulse driver. First of all, slew–rate limiting signals can reduce radiated emissions (electromagnetic interference (EMI) and radio frequency interference) and harmonics of its fundamental portion. Therefore, a digital signal, *f(t)*, that is not an ideal square wave, but may be approximated by a trapezoidal waveform with finite rise and fall times, *tr* and *tf*, can be employed for desired output signal analysis (Figure 1). This waveform exhibits characteristics of many other types of digital signals, including clock pulses and pulse width modulation (PWM) waveforms. In Figure 1, *A* stands for the amplitude of the signal, *Tperiod* is the signal’s period and *Ton* and *Toff* represent the turn-on and turn-off periods, respectively. The waveform can be expanded in a Fourier series, as shown in Equation (1), and the expansion coefficients are given by Equation (2) [25].

(1) $$f\left(t\right)=C_{0}+\overset{\infty }{\underset{n=1}{\sum }}C_{n}cos\left(n\omega_{0}t+\emptyset_{cn}\right)$$

(2) $$C_{n}=\frac{1}{T}\int_{t_{0}}^{t_{0}+T}f\left(t\right)e^{-jn\omega_{0}t}dt$$

where

(3) $$C_{0}=\frac{AT_{on}}{T_{period}}-\frac{A}{2}$$

For n≠0 and if $t_{r}=t_{f}$, the magnitudes of the Fourier coefficients, *Cn*, are given by

(4) $$C_{n}=2\frac{AT_{on}}{T_{period}}\left|\frac{\sin\left(\frac{n\pi t_{r}}{T_{period}}\right)}{\frac{n\pi t_{r}}{T_{period}}}\right|\left|\frac{\sin\left(\frac{n\pi T_{on}}{T_{period}}\right)}{\frac{n\pi T_{on}}{T_{period}}}\right|$$

From Equation (4) it can be observed that the waveform’s harmonic energy at high frequencies is less than that of an ideal square wave if the *tr* and *tf* are finite. Moreover, if the pulse has equal duty cycles for the turn-on and turn-off periods, that is,

(5) $$T_{on}=T_{off}=\frac{1}{2}T_{period}$$

and

(6) $$\left|\frac{\sin\left(\frac{n\pi T_{on}}{T_{period}}\right)}{\frac{n\pi T_{on}}{T_{period}}}\right|=\left|\frac{\sin\left(\frac{1}{2}n\pi \right)}{\frac{n\pi }{2}}\right|$$

*Cn* is equal to zero for even n, which stands for no even harmonics when the duty cycle of the excitation pulses is 50% (this is a reasonable assumption and can be easily achieved by tweaking the duty cycles of a trapezoidal-like waveform). The value of *tr* in Equation (4) affects the higher-order terms of *Cn*. Basically, the larger the *tr* or *tf*, the lower the harmonic terms at high frequency, which, in fact, has a low-pass filtering effect. Another issue that causes excessive harmonics is due to the difference between the rising and falling edges of such signals. When deriving the spectrum of this pulse train in Equation (4), equal *tr* and *tf* is assumed. However, even if the duty cycle is kept at 50%, large even harmonic signals still appear at the output of the pulse signals due to the inconsistency between the *tr* and *tf*.

**Figure 2:**
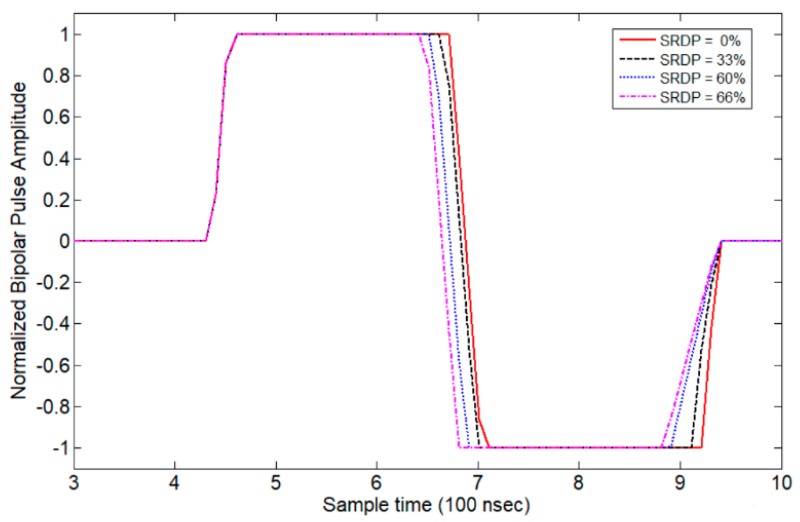
Simulated bipolar pulse waveforms with different SRDP between 0% to 66%.

To simulate the higher harmonic terms caused by unbalanced rising and falling edges, a repetitive trapezoidal waveform with a limited slew-rate and normalized −1.0 V to +1.0 V peak-to-peak voltage can be used to illustrate the effects. As shown in Equation (7), the effects of different slew-rates can be readjusted by changing the boundary conditions of each section. The simulated bipolar pulse signal with unbalanced slew-rates is plotted in Figure 2, which shows a general phenomenon when using a switching semiconductor device to generate such a waveform. These semiconductor devices typically exhibit a non-linear on-resistance, resulting in asymmetry in the rise and fall times of the output waveform. The pulses in Figure 2 assume uniform amplitude envelopes. The bandwidth of such waveforms is inversely proportional to the time duration. The center frequency of the signal is set to 2 MHz.

(7) $$f_{trapezoid}\left(x\right)=\{\begin{array}{cc} -4-\frac{4x}{\pi } & \mathrm{if}\;-\pi \;\leq x<\;-\frac{3\pi }{4}\\ -1 & \mathrm{if}-\frac{3\pi }{4}\leq x<\;-\frac{\pi }{4}\\ \frac{4x}{\pi } & \mathrm{if}\;-\frac{\pi }{4}\leq x<\;+\frac{\pi }{4}\\ +1 & \mathrm{if}+\frac{\pi }{4}\leq x\;<\;+\frac{3\pi }{4}\\ 4-\frac{4x}{\pi } & \mathrm{if}\;+\frac{3\pi }{4}\leq x\;<\;+\pi \end{array}$$

In Figure 2, the slew–rate difference in percentage (SRDP) of the Rising_time and Falling_time can be defined in Equation (8) as an important factor in evaluating signal harmonic leakage. In Equation (8), Rising_time and Falling_time are defined as the pulse signal responses to rise/fall from 10%/90% to 90%/10% of its final values, respectively.

(8) $$\mathrm{SRDP}=\frac{\left|\mathrm{Rising}\_\mathrm{time}\;-\mathrm{Falling}\_\mathrm{time}\right|}{\mathrm{Falling}\_\mathrm{time}}x100\%$$

**Figure 3:**
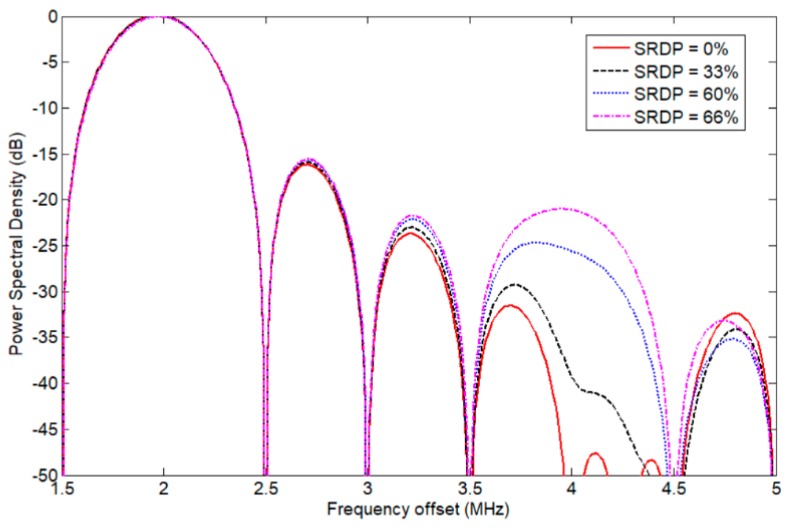
The frequency spectra of the bipolar pulse waveforms with different SRDP.

Each of the trapezoidal waveforms shown in Figure 2 can be mixed with the impulse response of the ultrasonic transducer and subjected to Fourier analysis to obtain the harmonic amplitude of the output waveform, as shown in Figure 3. A unit–gain ultra–wideband transducer is assumed here without loss of generality. The spectral differences between the signals shown in Figure 3 have equal turn-on/turn-off times, but different rising/falling slew-rates, and it is clearly indicated that the second harmonic component of the pulse waveform changes greatly due to the unbalanced slew–rates. For instance, the desired SRDP should be less than 33% in order to keep the second harmonic signal amplitude 40 dB lower than the fundamental one. Therefore, in order to generate an excitation signal with a low second harmonic content, equal rising and falling edges of the output pulses have to be carefully coordinated for the ultrasonic actuators.

## 3. Single-Chip High-Voltage Driver for Biomedical Ultrasound

**Figure 4:**
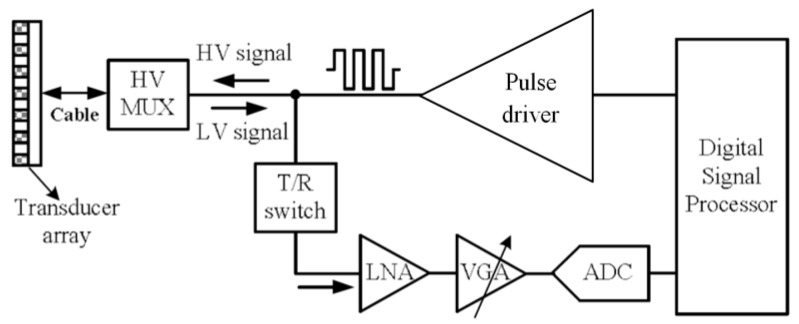
A typical biomedical ultrasound actuator/receiver system (HV MUX presents high-voltage multiplexer, LV and HV signals present low-voltage reflected and high-voltage excited signals, respectively, T/R switch presents the transmit/receive switch, LNA is the low noise amplifier, VGA is the variable gain amplifier, and ADC is the analog-to-digital converter).

A biomedical ultrasound image is acquired by transmitting acoustic waves and receiving echoes that are reflected from cell boundaries [1,2]. Figure 4 shows a typical biomedical ultrasound actuator/receiver system that comprises a pulse driver, a high-voltage multiplexer (HV MUX), a transmit/receive (T/R) switch, a low noise amplifier (LNA), a variable-gain amplifier (VGA) and an analog-to-digital converter (ADC) [16,17,18]. The digital signal processor generates thousands of properly delayed, low-voltage transmit patterns to the pulse drivers, which are converted into high-voltage signals (HV signals) to excite the transducers. These pulse-excited transducer arrays are used to generate a focused acoustic transmission. Then, due to the discontinuity of the acoustic impedance, the acoustic energy generated by the reflected wave is received by the transducer and converted into an electrical signal (LV signals) and sent to the LNA through the T/R switch. The HV MUX is designed to multiplex the transmit and receive signals to and from multiple piezoelectric transducers in the system, while the T/R switch is used to protect the LNA. When the driver is sending high–voltage transmit pulses, the LNA is isolated from the transducer by turning off the T/R switch. When the system is on the receive mode, the driver is disabled and the T/R switch is turned on. The LNA and VGA amplify the received echo signal and send the amplified signal to the ADC. The digital signal processor generates 2D and pulsed wave/color–flow Doppler information from the output data of the ADC [18]. In order to increase the strength of the echo signal, multi-channel transceivers are often indispensable in modern biomedical ultrasound systems, and they inevitably increase system complexity and power budgets. The generation of multiple high–voltage pulses, however, has been a challenge for ultrasonic imaging systems, particularly today’s portable systems. In the following section, we focused on the design and implementation of an integrated high-voltage pulse driver for multi-channel biomedical ultrasound actuators.

**Figure 5:**
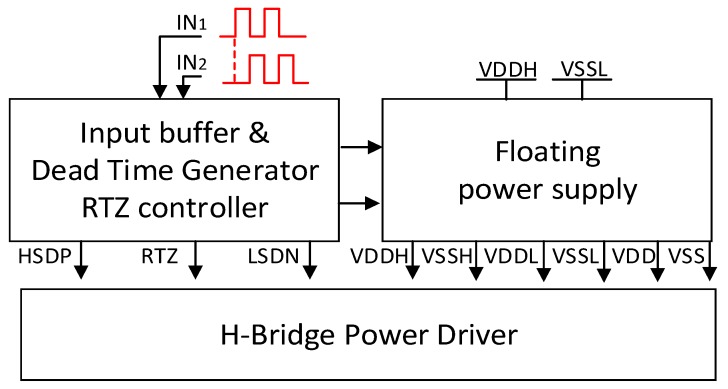
Proposed architecture of the integrated bipolar pulse driver (VDDH is the highest voltage, while VSSL is the lowest voltage supplied to the system, RTZ presents the return-to-zero control signal. HSDP and LSDN present the high-side and low-side control signal, VDD sets 5V and VSS sets -5V, and VSSH and VDDL are set by VDDH and VSSL, respectively, as defined in Equations (9) and (10)).

The schematic of the integrated high-voltage pulse driver is shown in Figure 5. It mainly consists of three stages, including the input stage, on-chip floating power supplies and H-bridge power driver. Several techniques are employed in this high-voltage pulse driver design to achieve low static power and low harmonics at the output. These techniques are described in the following section.

### 3.1. Input Stage

**Figure 6:**
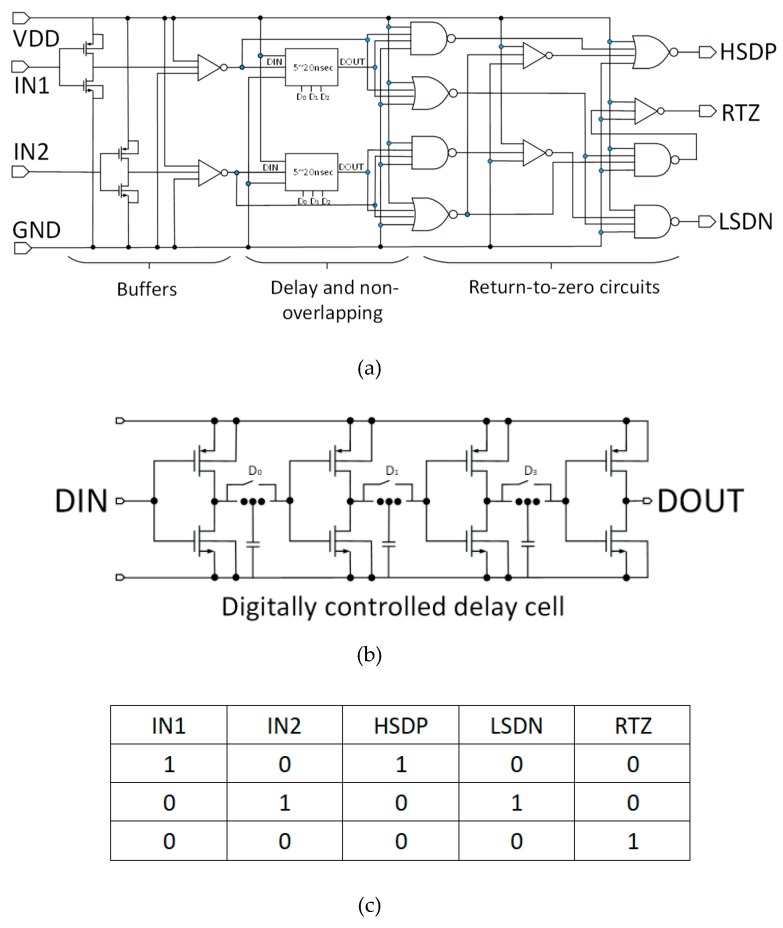
Input stage of the bipolar pulse driver (**a**), the digitally controlled delay element (**b**), and the truth table of the control signal versus the inputs (**c**), where DIN and DOUT presents the control signal fed before and after the delay element.

The input stage, as shown in Figure 6a, consists of signal conditioners (buffers), the delay element and a return-to-zero signal generator. The input stage processes the input signal fed from the outside signal generator (i.e., the Field Programmable Gate Array (FPGA)) controller and produces three signals (i.e., HSDP, LSDN, and RTZ as shown in Figure 5) to switch the H-bridge power driver on and off. Since the input signals IN1 and IN2 work independently to control the H-bridge, a non-overlapping circuit inside the pulse driver system was designed to avoid any possible current shoot-through between power transistors. In order to achieve this, a 3-bit digitally adjustable delay element was designed to control the timing gap of the non-overlapping circuit (Figure 6b). The truth table of the output driving signal vs the input signal is shown in Figure 6c. 

### 3.2. H-Bridge Power Driver

**Figure 7:**
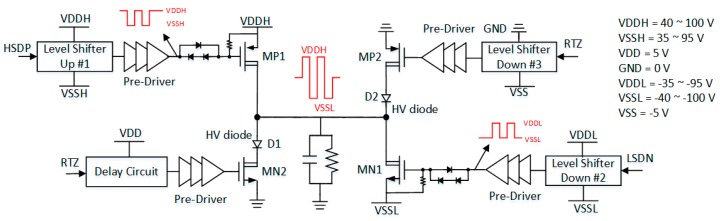
The H-bridge power driver and its slew-rate controller.

**Figure 8:**
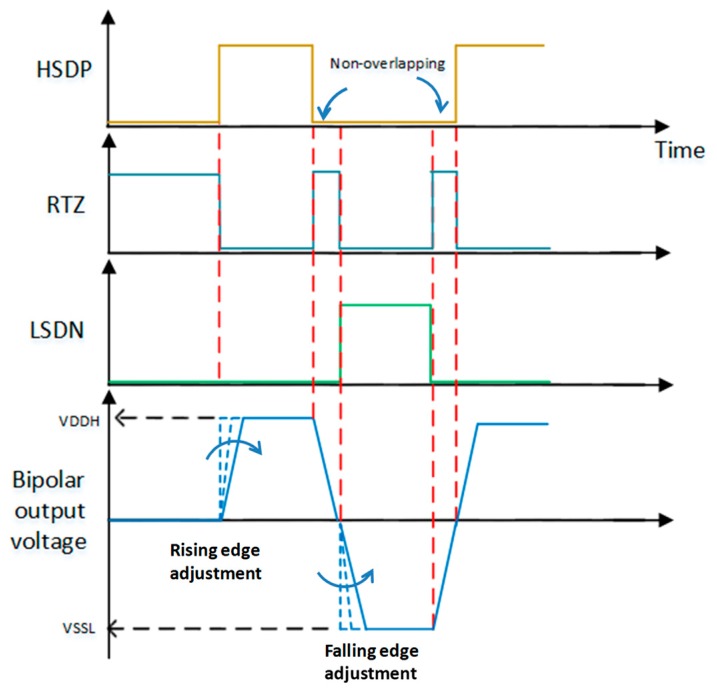
The schematic of the output pulse sequences and the rising/falling edge adjustment.

The H-bridge power driver consists of level-shifters, pre-drivers, two high-voltage (HV) diodes (D1/D2) and four power FETs (MP1/2 and MN1/2), as shown in Figure 7. The two signals, HSDP and LSDN, are used to turn on MP1 and MN1 by employing two floating level-shifters, Level-Shifter-up #1 and Level-Shifter-down #2, as well as pre-drivers to boost their driving voltage levels to within the voltage level of VSSH to VDDH and VDDL to VSSL, respectively. When the ultrasound system is in the receive mode, the RTZ signal turns on MP2 and MN2 and returns zero voltage from either the VDDH or VSSL voltage level to avoid affecting the reception of the echo signals. In order to adjust the turn-on dV/dt rate of the final-stage power FETs, diodes are placed in a series between the gates of MP1/MN1 and the pre-drivers. The diodes set the turn-on voltage and hence can be used to adjust the overall turn-on rising edge of the power FETs. Another advantage is the ability to reduce EMI or circuit switching noise. Figure 8 shows the schematic of the turn-on sequences and turn-on dV/dt slew-rate adjustment of the H-bridge. On the other hand, the size of the HV diodes and MP2/MN2 set the return-to-zero speed of the H-bridge, which should be set comparable to the turn-on rising edge of MP1/MN1. The size selection for four power FETs will be described in detail in Section 3.5. 

### 3.3. Low-Power Floating Power Supply 

**Figure 9:**
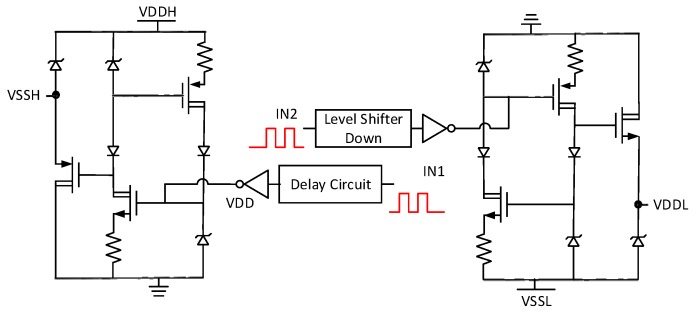
Floating power supply schematic and its controller.

Since the output stage uses push–pull architecture, a pair of high-voltage power devices are employed as the output stage. The design of their driver stages can also use push–pull architectures to efficiently drive the output stage. Since the gate voltage of high-voltage PMOS/NMOS devices is limited by the maximum operating voltage of Vgs, the output voltage swing of the driver has to be within 5 V. Therefore, each driver requires a set of 5-V rail-to-rail power supplies to provide a source/sink current to the output. In order to simplify the power supplies, a floating voltage source architecture is employed. Figure 9 shows two floating voltage sources generated by the VDDH and VSSL to ground, respectively. The pair of high-voltage PMOS/NMOS devices in series with two sets of Zener and high-voltage diodes form a voltage divider loop and output two voltages respective to the voltage drop of the Zener diode. Another two high-voltage PMOS/NMOS devices in parallel with the divider loop are functioned as the source follower to provide stable output voltages. For instance, the output voltages VDDL and VSSH are generated by VSSL and VDDH, as shown in Equations (9) and (10), respectively.

(9) $${\mathrm{VSSH}=\mathrm{VDDH}-V}_{\mathrm{Zener}}$$

(10) $${\mathrm{VDDL}=\mathrm{VSSL}+V}_{\mathrm{Zener}}$$

One of the advantages of this architecture is its fast settling and stable output, even though the output voltage is not accurately regulated. However, it dissipates power once the VDDH or VSSL is applied, which degrades the efficiency of the overall system. Therefore, switch control signals generated by the inputs IN1 and IN2 are employed. The floating power supply can only work following the input signal’s commands. When there is no input signal for a period of time, the floating power supply can be turned off to reduce power dissipation.

### 3.4. Capacitor-Coupled Floating Level-Shifter Design

**Figure 10:**
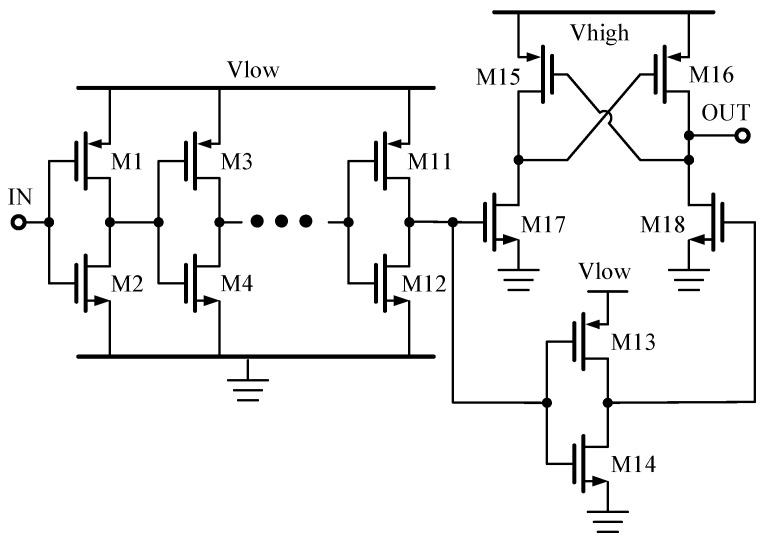
Schematic of a latch-based full-swing level-shifter.

Level-shifters are used in applications that require interfaces between different voltage domains. There are two types of level-shifters, full-swing and floating, which can be distinguished by whether the voltage domain shares a common ground potential. Figure 10 shows a schematic of a conventional latch-based full-swing level-shifter, which is used to boost the input signal from a “Vlow” level to a “Vhigh” level. The inverter chain (M1–M12) is used to reconstruct the rail-to-rail digital signal from the off-chip input control signal. The cross-couple pair (M15 and M16) can latch the “high” digital signal level. When the input signal switches to a “low” level, M16 and M17 are turned off, and M15 and M18 are turned on. The output voltage is 0 V. However, as the input signal becomes a “high” level, M16 and 17 are switched on, and M15 and M18 are switched off. The output voltage then becomes Vhigh. 

**Figure 11:**
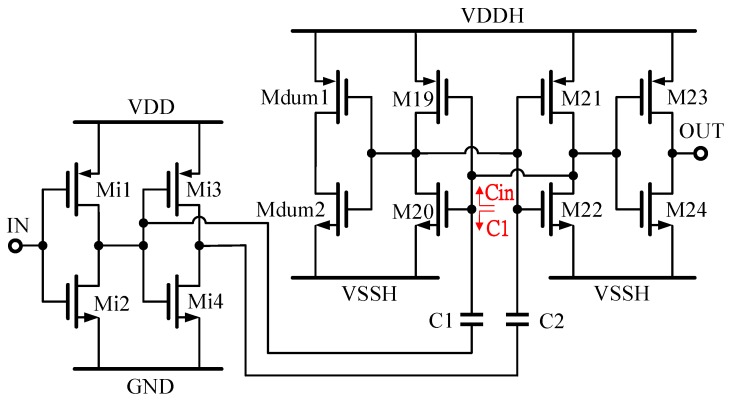
Schematic of a latch-based capacitor-coupled floating level-shifter.

The full-swing level-shifter shown in Figure 10 is not appropriate for power FET gate driver design since the gate driver needs a floating rail to switch on/off the final stage of the power devices. Floating level-shifters, however, can shift the potential of control signals from circuits of a low-voltage power rail to potential with floating and ground rails, and therefore floating level-shifters are often used in gate drivers to drive output stages. Figure 11 shows the designed capacitive-coupled level-shifter architecture employed for the H-bridge power driver. For instance, the Level Shifter #1 in Figure 7, is used to shift the signal voltage levels of 0 V and VDD to VSSH and VDDH, respectively. The level-shifter consists of a pair of inverters (Mi1–Mi4), two coupling capacitors (C1 and C2), a latch (M19–M22), an output inverter (M23–M24) and a dummy inverter (Mdum1 or Mdum2), which is utilized to keep the same output impedance seen by inverters of M21 and M22. The pair of inverters (Mi1–Mi4) operates at a 5-V power supply, while the latch, dummy inverter and output inverter are supplied by VSSH and VDDH power sources. Mi1 and Mi4 and the latch are isolated by two coupling capacitors, C1 and C2, which couple through the control signals to the output stages. 

**Figure 12:**
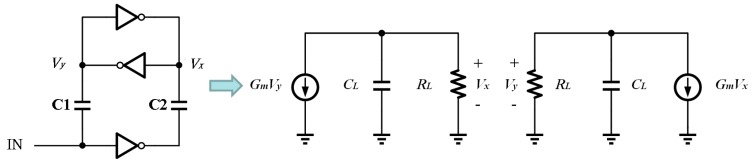
Simplified modeling of the floating level-shifter.

Figure 12 shows the simplified model of the latched stage with capacitor coupling between the input and the leveled outputs, where *Gm* represents the sum of the trans-conductance of M19 and M20 (same as M21 and M22), *CL* is the input capacitance of M23 and M24 and *RL* represents the output node impedance of M19 and M20. Following Figure 11, the dynamic behaviors of the latch outputs, *Vx* and *Vy*, can be written into Equations (11) and (12).

(11) $$G_{m}V_{y}=-C_{L}\left(\frac{dV_{x}}{dt}\right)-\left(\frac{V_{x}}{R_{L}}\right).$$

(12) $$G_{m}V_{x}=-C_{L}\left(\frac{dV_{y}}{dt}\right)-\left(\frac{V_{y}}{R_{L}}\right).$$

By replacing *RL* and *CL* with τ = *RLCL*, *Av* = *GmRL*, and reordering the formula, Equations (13) and (14) represent the cross-correlation between *Vx* and *Vy*.

(13) $$\tau \left(\frac{dV_{x}}{dt}\right)+V_{x}=-A_{v}V_{y}.$$

(14) $$\tau \left(\frac{dV_{y}}{dt}\right)+V_{y}=-A_{v}V_{x}.$$

Using Equations (13) and (14) we can solve

(15) $$\delta V=\delta V_{0}*e^{\frac{\left(A_{v}-1\right)t}{\tau }}$$

where *δV* is the voltage difference between the input and output of the Latch (i.e., *Vx*-*Vy* in our design), and $\delta V_{0}$ is the initial voltage difference at the beginning of the latch phase. Based on Equation (15), the transition time of the latch can be solved as expressed in Equation (16).

(16) $$T_{latch}≅\frac{C_{L}}{G_{m}}*ln\left(\frac{\delta V}{\delta V_{0}}\right)$$

The rising/falling slew-rate of the level-shifter,$SR^{+/-}$, can then be defined as

(17) $$SR^{+/-}=\frac{\delta V}{T_{latch}}$$

**Figure 13:**
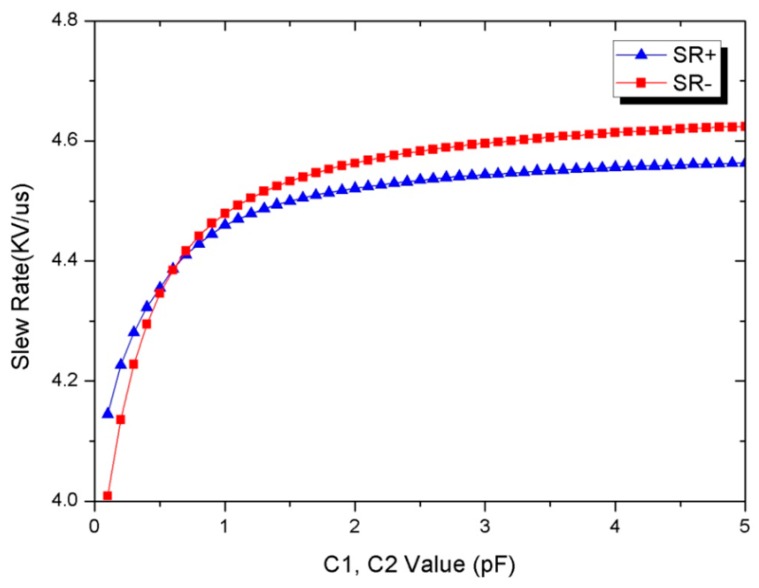
Simulated slew-rate of rising and falling edge of the level-shifter.

**Figure 14:**
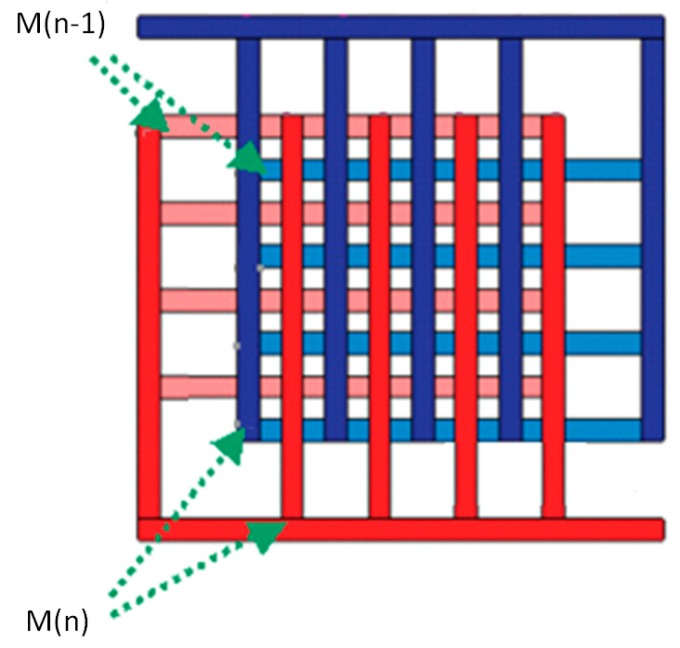
Schematic of the on-chip metal-oxide-metal capacitor (M presents the layer of the overlapping fingers).

Since the latched time is reversed-logarithmic proportional to $\delta V_{0}$, *Tlatch* will be too large to affect the desired slew-rate if $\delta V_{0}$ is a small value. From Figure 11, we can find $\delta V_{0}=\frac{C_{1}}{C_{1}+C_{in}}\mathrm{VDD}$. The ratio between the coupling capacitance and the input capacitance of the latches affects the latched time. The value of C1 can therefore be designed comparably to that of Cin in order to avoid a small $\delta V_{0}$, as shown in Figure 11. In addition, in order to have a balanced slew-rate of the level-shifters, the coupling capacitance ratio between C1 and C2 has to be investigated to ensure the slew-rate performance between these level-shifters once the latches have been designed. Figure 13 shows that the simulated slew-rate of the level-shifter as the value of the coupling capacitance is varied accordingly. The optimal design can be found by choosing the corresponding coupling capacitance with *SR*+ equal to *SR*−. Since the coupling capacitor has to withstand a large voltage drop between VDDH and VDD, on-chip metal-oxide-metal (MOM) capacitors are employed in series to increase the voltage resistance to 100 V for such applications. Figure 14 shows the schematic diagram and layout of the on-chip MOM capacitor.

### 3.5. Final Stage Power Inverter Design

**Figure 15:**
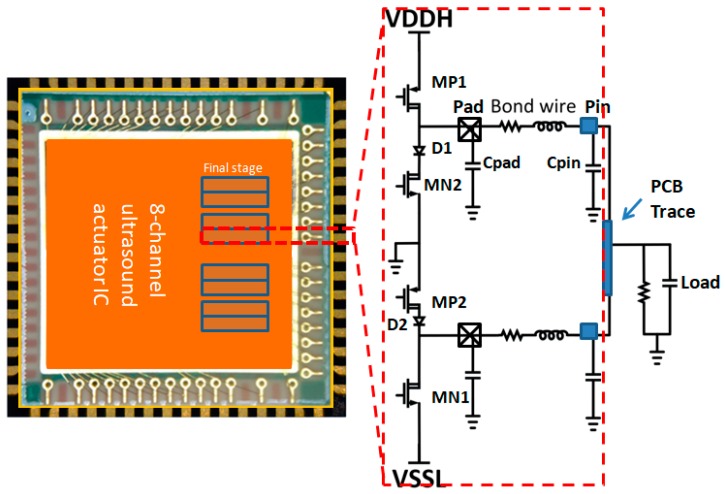
Package/chip joint design with parasitic parameter extractions.

**Figure 16:**
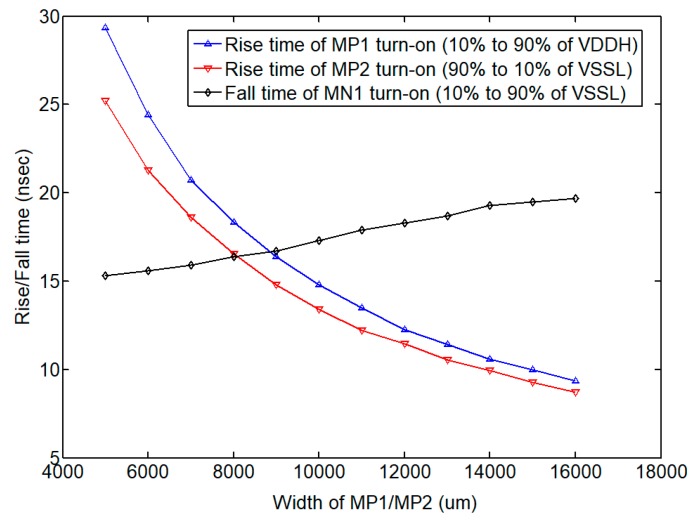
Simulated rise/fall time versus the final-stage power FET device sizes.

In addition to considering the size of the transistor providing the load driving capability, the parasitic effects of the package and bonding wires were also attended in the design phase to design the output power FETs,. Figure 15 shows the outline of the QFN-64L package for the eight-channel ultrasound pulse driver IC. The equivalent parasitic parameters Cpad, Cpin, Rb and Lb extracted by Ansys Q3D extractor (ANSYS Inc., Canonsburg, PA, USA) are 0.4 pF, 1 pF, 0.4 Ω and 1 nH, respectively. These parameters, along with the equivalent load impedance, participate in the design to determine the size of the power FETs. As described in Section 3.2, the size ratio between the MP1/2, MN1/2 and the HV diodes determines the turn-on/turn-off time of the high-voltage H-bridge. In our design, the dimensions of the transistors MP1 and MP2 are swept accordingly to account for the external slew-rate of the final stage, which is based on the fixed size of MN1 shown in Figure 15. Since the output current capability of the power FET is approximately proportional to the transistor size, the current capability of the output node becomes stronger and the charging rise-time can become shorter as the size increases. However, as the transistor size gets too big, the excessive capacitance at the output node increases the discharge time. Therefore, the size selection of the power transistors must seek an appropriate dimension for the power transistors, as the rise and fall times can overlap (i.e., the rise and fall times become almost equal). Figure 16 shows the simulated rise/fall times (from 10% to 90% VDDH or VSSL) versus the size of MP1 and MP2, respectively. The best design for this application is to set a width of approximately 9500 μm for MP1 and 8000 μm for MP2 in order to allow the final-stage power FETs to operate at the similar slew-rate. To ensure the quality of the final output, corner and Monte Carlo simulations were performed during the design phase with a special emphasis on the effect of the output stage size. During the layout phase, we set several adjustment points inside the circuit to reduce the impact of process-voltage-temperature (PVT) on overall performance. Table 1 summarizes the optimized dimensions of each power transistor in this design.

## 4. Experimental Results and Discussions

**Figure 17:**
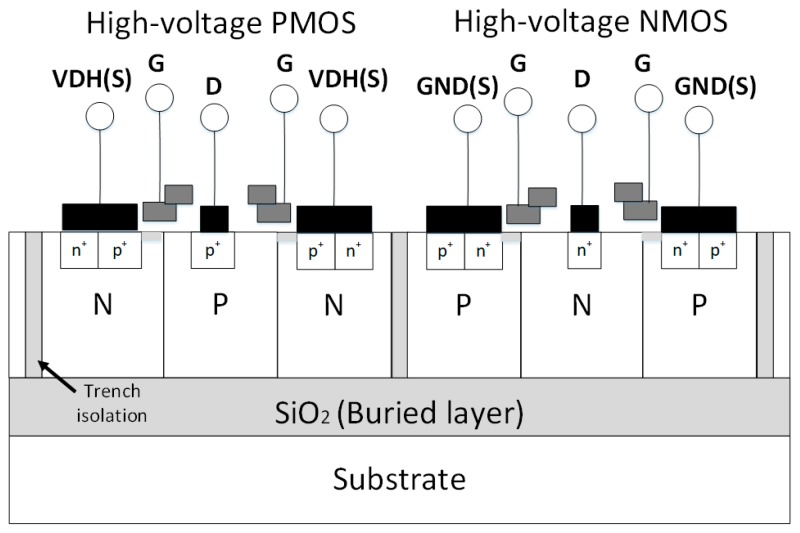
The cross-section view of high-voltage (HV) devices in a CMOS-SOI process.

**Figure 18:**
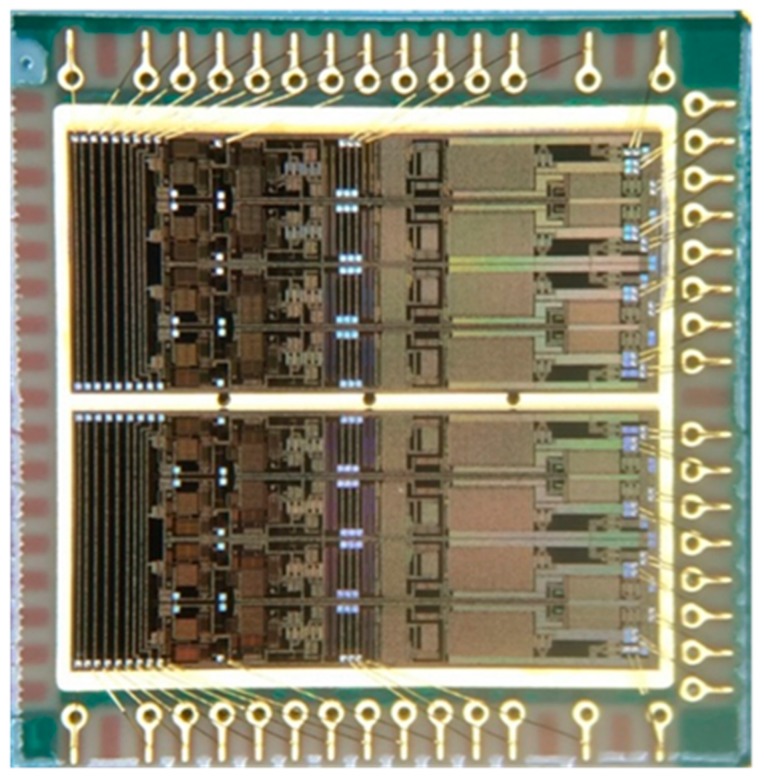
The photo of the integrated eight-channel high-voltage pulse driver.

The high-voltage pulse driver was fabricated in a 0.5 μm CMOS-SOI technology, which allows mixing different structures such as CMOS for digital circuits and high-voltage MOS structures for power and high-voltage applications on the same wafer with buried isolation layer [26]. Figure 17 shows a cross-section of the CMOS-SOI process, and the high-voltage MOS transistors are fully compatible with the existing CMOS process. The final stage power transistors used in the design are 150-V N- and P-channel drain-extension field-effect transistors (FETs). The chip micrograph of the designed eight-channel pulse driver is shown in Figure 18, and it measures 8000 μm × 7100 μm area. The performance verification of the high-voltage pulse driver was carried out by electrical and acoustic field measurements, which will be introduced in the following section.

### 4.1. Electrical Performance Verification

**Figure 19:**
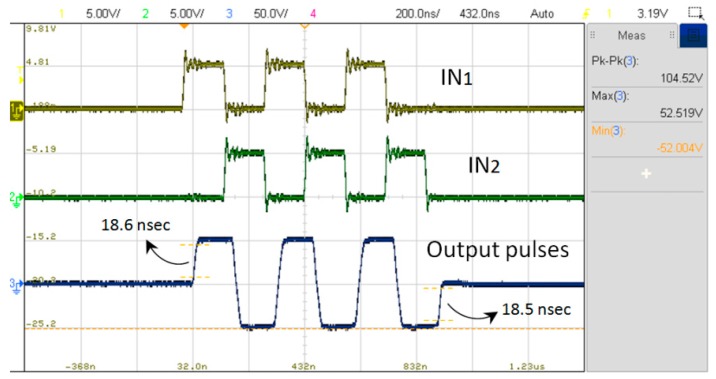
Measured input signals and output pulses of the designed driver under a 1-K ohm resistance in parallel with a 220-pF capacitance load.

**Figure 20:**
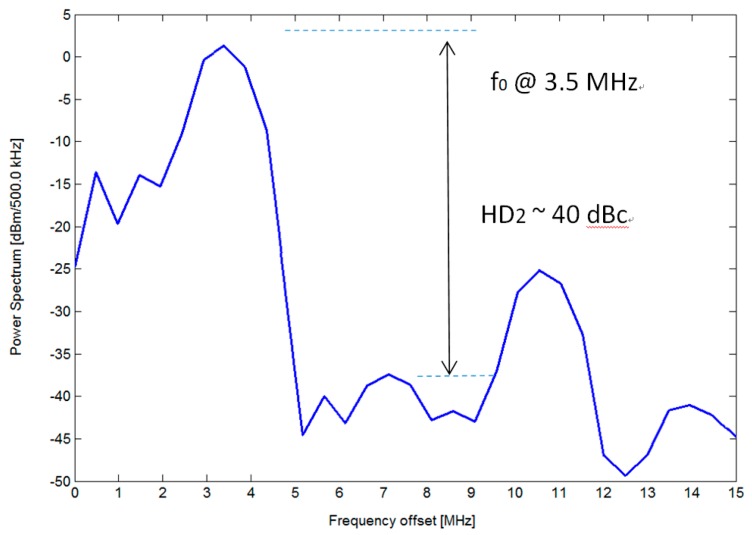
Spectrum analysis of the measured output pulses in Figure 19.

The electrical characteristics of the designed high-voltage ultrasound pulse driver were verified using a gated input signal at 3.5 MHz and dummy loads with a 1-K ohm resistance in parallel with a 220-pF capacitance. Figure 19 and Figure 20 show the measured output bipolar voltage waveform and its spectrum diagram, respectively. The output voltage can reach more than 100 Vpp with rising and falling times of 18.6 and 18.5 nsec. The second harmonic distortion is down to −40 dBc. Table 2 records that the static power consumption of a single channel pulse driver of approximately 3.6 mW (including the power consumption of 11.24 μW from the leakage of the floating power supply) without a drive signal input (VDD = 50 V, VSS = −50 V). The power consumption of the driver increases slightly with the switching frequency of the input drive signal. Since the floating power supply is triggered by the input signal and is intended to supply the eight-channel pulse drivers, it consumes about 100 μA of static DC current. The power consumed by each driver is approximately 48~49 mW when the excitation period of the high-voltage pulse signals accounts for 1% of the pulse repetition frequency (PRF = 10 kHz). Table 3 summarizes a performance comparison with several published works. Compared to the other more complex circuit architectures in Table 3, the proposed pulse driver achieves the same operating frequency and rise/fall slew-rates (>3.5 kV/μsec), while the HD2 performance is also less than −40 dBc, meeting the requirements for harmonic imaging applications as described in Section 2. In addition, there are two aspects of performance that are more prominent than the earlier works. First, the proposed architecture designs floating voltage sources inside the chip that can greatly reduce the number of voltage sources required externally. Moreover, the floating power supply activated by the input control signal can reduce the overall chip power dissipation, thereby reducing the input power of the driver without affecting the output drive capability. This is very important for portable ultrasound scanners. The lower the power consumption per channel, the more channels the system can use to excite the ultrasonic actuator with a fixed input power, which helps to improve the overall quality of the ultrasound image.

### 4.2. Acoustic Field Measurement Results

**Figure 21:**
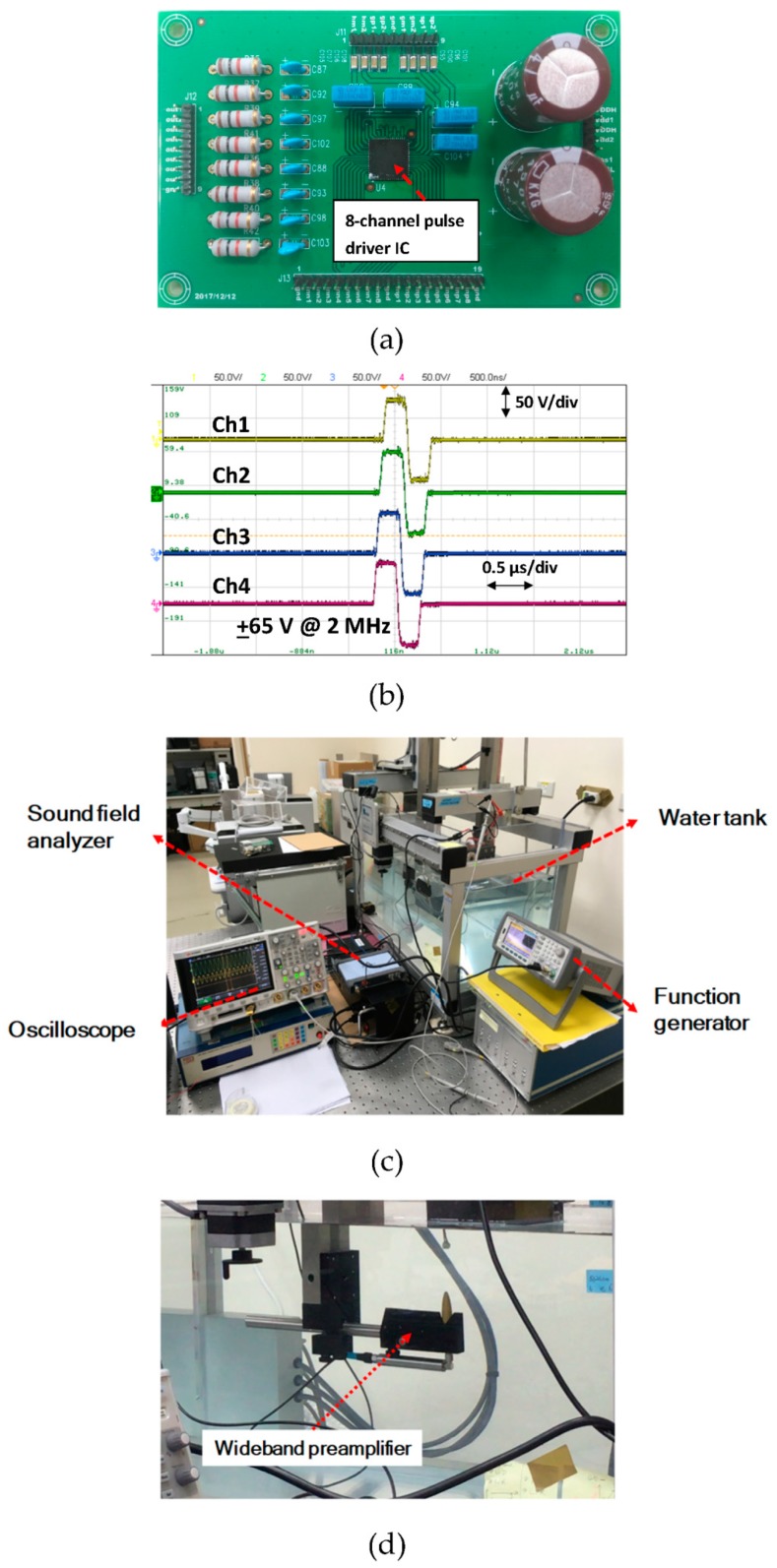
The eight-channel pulse driver integrated circuit in a single package and its assembly (**a**), multiple-channel delayed high-voltage pulses (**b**), the Acoustic Intensity Measurement System (AIMS) and the associated equipments (**c**) and the wideband receiver of the sound field measurements (**d**).

The integrated eight-channel ultrasound pulse driver test board for verifying transmit beamforming was assembled and tested with a phased array probe. The probe under test was BS7L3 made from Broadsound corporation (Hsinchu, TW), which has a fractional bandwidth of over 60%. The experiment was performed using the Acoustic Intensity Measurement System (AIMS) made from Onda corporation (Sunnyvale, CA, USA). Figure 21a shows a picture of the designed eight-channel driver test board and Figure 21b shows the high-voltage output waveform of four of the channels. The measurement setup, including a water tank to model the underwater environment and a sound file analyzer to synthesize the beamforming results, is shown in Figure 21c. After generating the input beamforming signals of different delay times by FPGA encoding, transducers were excited by these eight sets of high-voltage pulses. A wide dynamic range preamplifier was used to measure the sound field produced by the ultrasound probe after excitation by the actuators. Figure 21d presents the wideband receiver for the acoustic field measurements.

**Figure 22:**
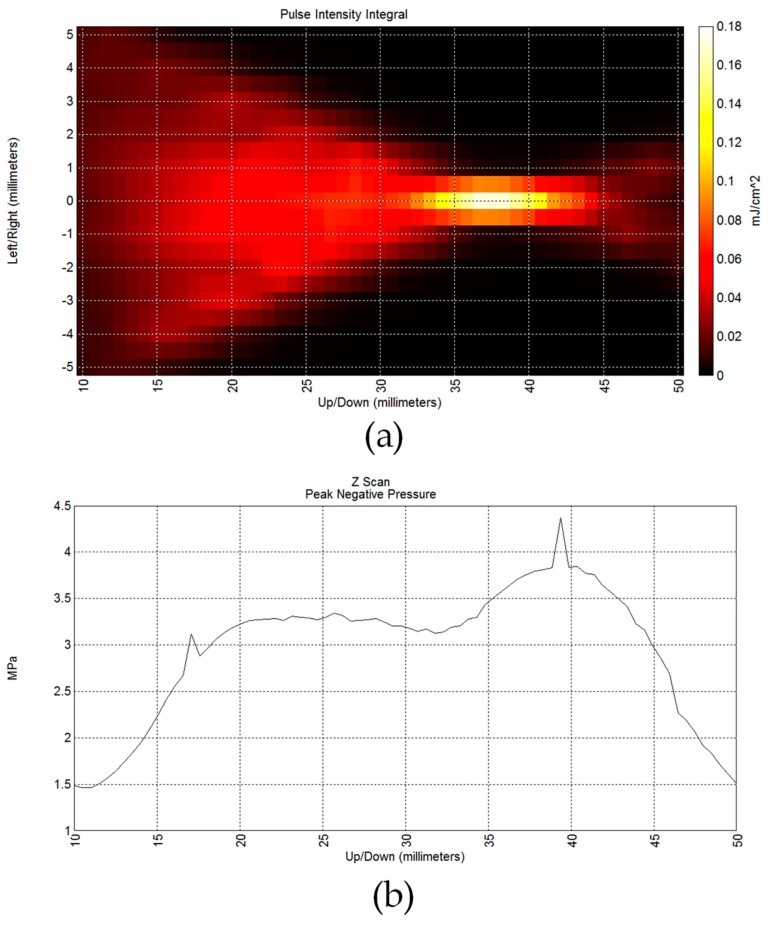
Measured underwater acoustic field using the eight-channel pulse actuator (**a**). The maximum 4 MPa was obtained at the focal plane after beamforming (**b**).

Figure 22 shows the measurement results of the transmit beamforming. The maximum 4 MPa was obtained at the focal plane after beamforming. At a focal plane 3.5~4.0 cm away from the probe, the beam intensity is about 180 μJ/cm^2^.

**Figure 23:**
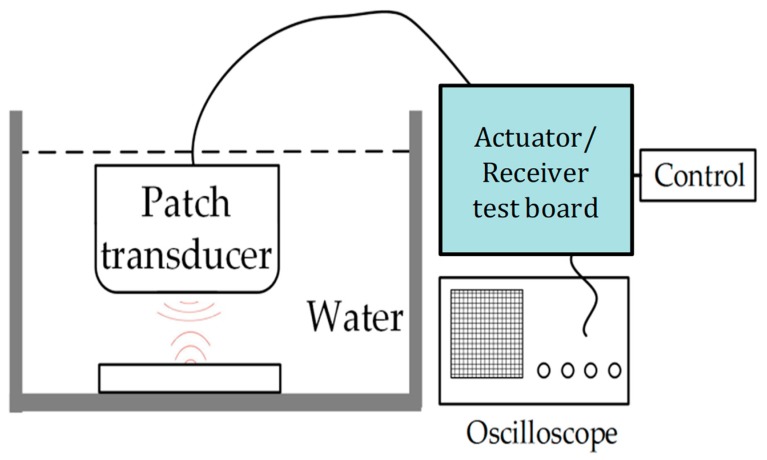
The measurement setup for underwater echo signal acquisition using the actuator/receiver system.

**Figure 24:**
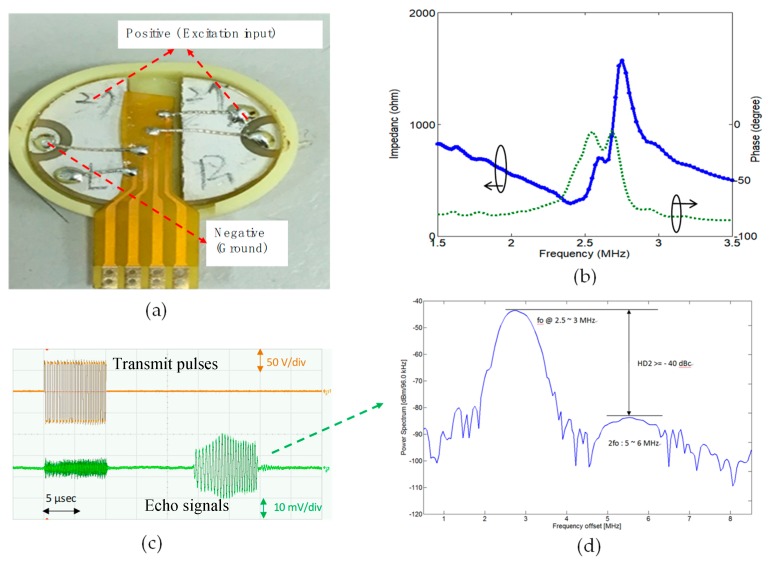
The actuator/receiver harmonic leakage verification measurement with a patch transducer as the load of the actuator (**a**), the impedance measurement of the patch transducer (**b**), high-voltage bipolar pulses as transmitting signals (orange) and receiving signals (green) (**c**) and the spectrum analysis of the echo signals (**d**).

To verify the harmonic content of the echo signal, another measurement setup was performed using a single-channel patch transducer with the designed driver. Figure 23 shows the measurement setup. The patch transducer can act as both an excitation and an echo signal acquisition device. Figure 24a shows the picture of the patch transducer, and the impedance measurement result is shown in Figure 24b. The high-voltage pulse waveform for excitation and the echo signal measured by the transducer are shown in Figure 24c. The spectrum obtained by Fourier analysis of the echo signal is shown in Figure 24d. The second harmonic leakage at the receiver compared to the fundamental signal was less than 40 dB, indicating that this ultrasound driver meets the basic criteria for harmonic imaging applications.

## 5. Conclusions

The designed high-voltage pulse driver, which includes a high-voltage H-bridge driver with two pairs of P/N high-voltage MOSFETs and diodes associated with high-speed capacitor-coupled level-shifters and an input stage for the signal conditioner, can provide a low second harmonic (−40 dBc), a high-voltage output (>100 Vpp) with a wide oscillation frequency (>10 MHz) and low power consumption (<3.6 mW/per channel). In addition, a dual input signal control floating power supply simplifies the design of integrated ultrasound systems. The overall performance of the eight-channel high-voltage bipolar pulse driver was electrically and acoustically verified by the ONDA sound field measurement system, respectively. The experimental results indicate the proposed design has high potential for medical ultrasound scanners, especially for advanced tissue harmonic imaging applications.

## Figures and Tables

**Table 1:** Size selections for the finals stage power transistors and HV diodes.

Power Device Name	MP1	MN2	MN1	MP2	D1/D2
Width (μm)	950 × 10	250 × 11	300 × 11	800 × 10	100 × 8
Length (μm)	0.5	0.9	0.9	0.5	36

**Table 2:** Measured power consumption of the ultrasound pulse driver per channel in the excitation mode (excitation rate = 1% of the pulse repetitive frequency).

Input Signal Frequency.	No input (Stand-by)	3.5 MHz Pulse	7.5 MHz Pulse	10 MHz Pulse
VDDH = 50 V and VSSL = −50 V	3.6 mW	48.6 mW	48.8 mW	49.2 mW

**Table 3:** Measured-performance summary and comparison.

	This Work	[23]	LM96550 [21]	MAX14808 [19]	MD2130 [22]
Voltage level	3	3	3	3/5	Continuous
External supply voltage sources	4	6	6	5/7	5
Technology	0.5 μm CMOS*-SOI	0.25 μm BCD+	N/A	N/A	CMOS* (discrete)
Output voltage (V)	0 to ±75	0 to ±40	0 to ±50	0 to ±100	0 to +125
Channel no. Maximum frequency (MHz)	8up to 10	1 up to 20	8 up to 15	8 up to 10	1 up to 15
Peak current (A)	2	2	2	2	3
Rising time (nsec)	18.6	8.6	18	21	-
Falling time (nsec)	18.5	8.5	18	21	-
Slew rate (V/μsec)	3.7 K	4.7 K	2.2 K	3.8 K	-
Output load	1 kΩ// 220 pF	100 Ω//100 pF	100 Ω// 330 pF	1 kΩ// 240 pF	1 kΩ// 220 pF
HD2 (dBc)	−40	−40	−40	−43	−46
Standby power (mW/ch)	<4	5.5	32	17	152
Working power (mW/ch)	<50	-	-	73	-

CMOS*: Complementary metal-oxide-semiconductor; BCD+: Bipolar-CMOS-DMOS.
